# Spirochete Flagella and Motility

**DOI:** 10.3390/biom10040550

**Published:** 2020-04-04

**Authors:** Shuichi Nakamura

**Affiliations:** Department of Applied Physics, Graduate School of Engineering, Tohoku University, 6-6-05 Aoba, Aoba-ku, Sendai, Miyagi 980-8579, Japan; naka@bp.apph.tohoku.ac.jp; Tel.: +81-22-795-5849

**Keywords:** spirochetes, periplasmic flagella, motility, chemotaxis, molecular motor

## Abstract

Spirochetes can be distinguished from other flagellated bacteria by their long, thin, spiral (or wavy) cell bodies and endoflagella that reside within the periplasmic space, designated as periplasmic flagella (PFs). Some members of the spirochetes are pathogenic, including the causative agents of syphilis, Lyme disease, swine dysentery, and leptospirosis. Furthermore, their unique morphologies have attracted attention of structural biologists; however, the underlying physics of viscoelasticity-dependent spirochetal motility is a longstanding mystery. Elucidating the molecular basis of spirochetal invasion and interaction with hosts, resulting in the appearance of symptoms or the generation of asymptomatic reservoirs, will lead to a deeper understanding of host–pathogen relationships and the development of antimicrobials. Moreover, the mechanism of propulsion in fluids or on surfaces by the rotation of PFs within the narrow periplasmic space could be a designing base for an autonomously driving micro-robot with high efficiency. This review describes diverse morphology and motility observed among the spirochetes and further summarizes the current knowledge on their mechanisms and relations to pathogenicity, mainly from the standpoint of experimental biophysics.

## 1. Introduction

Motility systems of living organisms are currently classified into 18 types [1]. Even when focusing on bacteria only, the motility is diverse when bacterial species are concerned [2]. A major motility form would be the flagella-dependent swimming well observed and described in *Escherichia coli* and *Salmonella enterica*, and these species have helical flagella extending to the cell exterior. Spirochetes, which are members of a group of gram-negative bacteria with a spiral or flat-wave cell body, also show flagella-dependent motility, but their flagella are hidden within the periplasmic space and are thus called periplasmic flagella (PFs). Externally flagellated bacteria are propelled by direct interaction of flagella and fluid, whereas spirochetes swim by rolling or undulation of a cell body driven by PFs rotation beneath the outer membrane. Physics difference results in an invalidation of applying the canonical model obtained from external flagella to spirochetal periplasmic flagella.

This review article describes the motility of spirochetes while connecting it with the unique structures of their cell bodies and PFs. Taxonomically, the phylum *Spirochaetae* is classified into *Leptospiraceae*, *Brachyspiraceae*, *Spirochaetaceae*, and *Brevinemataceae* families, containing pathogenic species, for example, *Leptospira interrogans* (leptospirosis), *Brachyspira hyodysenteriae* (swine dysentery), *Borrelia burgdorferi* (Lyme disease), and *Treponema pallidum* (syphilis). As observed with other motile pathogens, spirochete motility is an essential virulence factor. Thus, the last part of this review discusses the involvement of motility in spirochetal pathogenicity.

## 2. Cell Structure

**Figure 1:**
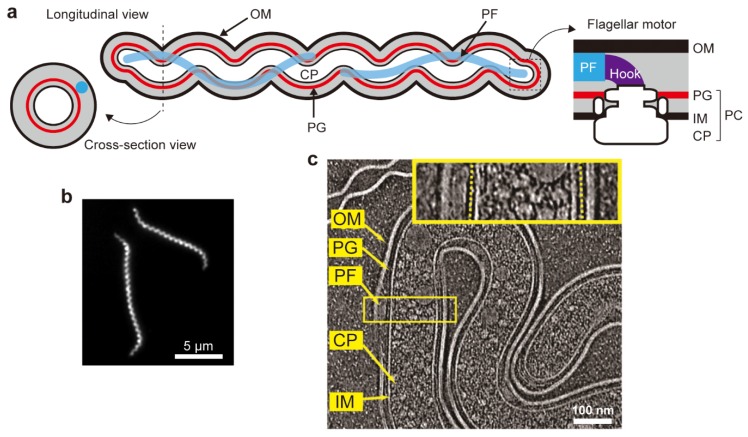
Spirochetal cell structure. (**a**) Schematics of longitudinal and zoom-in cross-section views of the cell structure and the flagellar motor shared by spirochete species; outer membrane (OM), periplasmic flagellum (PF), peptidoglycan layer (PG), inner membrane (IM), cytoplasm (CP), and protoplasmic cylinder (PC) are shown. If readers view from the hook to the motor, the flagellar motor rotates in a counterclockwise (CCW) direction at one pole of a single cell, whereas the motor at another cell pole rotates in a clockwise (CW) direction. (**b**) Dark-field micrograph of *Leptospira biflexa*. (**c**) Longitudinal slice image obtained by cryo-electron tomography of *L. biflexa* (adapted from [14] with permission from the publisher). OM, IM, and PF are clearly visible, and PGs observed in the yellow square are indicated by yellow dashed lines in the enlarged view (inset).

A schematic of the basic structure shared among spirochete species is shown in Figure 1a. The protoplasmic cylinder consists of a cytoplasm, a cytoplasmic membrane, and a peptidoglycan layer, which is covered by the outer membrane. Each PF filament connects with a basal motor called the flagellar motor that is embedded in the cytoplasmic membrane and the peptidoglycan layer via a short, bent structure corresponding to the universal joint hook in the *E. coli* flagellar motor (details are described below) [3]. The morphologies of the cell body and the PF as well as the number of PFs greatly differ among species, and those of three representative species are summarized in Table 1. The cell body of *Borrelia* spp. exhibits a flat-wave shape and contains 7~11 PFs long enough to overlap with those extending from the other end at the center of the cell body [4,5,6,7]. *Brachyspira* spp. appear to have a flat-wave body because of their non-spiral, almost straight configuration observed in swimming cells [8], but no explicit evidence has been reported. *Brachyspira* PFs overlap at the cell center, and so do those of *Borrelia* [9]. The cell morphology of *Leptospira* spp. is distinguished from the other two spirochetes by a small cell width and short wavelength [4,10]. The protoplasmic cylinder of *Leptospira* (Figure 1b,c) is relatively rigid, maintaining the helix parameters even during swimming, whereas both ends of the cell body are frequently transformed, as described later [11,12,13,14]. Unlike *Borrelia* and *Brachyspira*, PFs of *Leptospira* are too short to overlap [15]. 

## 3. Periplasmic Flagella

### 3.1. Physical Properties of the PF Filament

The flagellar filament of *E. coli* functions as a screw propeller through interaction with fluid [24]. In contrast, spirochete PFs are thought to rotate or transform the cell body by intimate contact with cell membranes, although direct observation of the PF rotation has not been successful. Another important role of the PF is to establish a wavy morphology, similar to a cytoskeleton, and the PF dependence of spirochete morphology has been observed in the periodontal disease-associated spirochetes *Treponema denticola* [25], *B. burgdorferi* [26,27], and *Leptospira* spp. [15,19,20,21,22]. For example, the loss of the PF in *B. burgdorferi* straightens the entire cell body [26]. In contrast, *Leptospira* PF depletion affects only the bent morphology of the cell ends, and the short-pitch helix in the protoplasmic cylinder is believed to be maintained by a bacterial actin homolog, MreB [28]. Both the cell body and the PF can be considered elastic materials, and the observed PF-dependent spirochete morphology is a consequence of the mechanical interaction between these two elastic bodies of different stiffness [29,30]. This difference in stiffness between the cell body and the PF can be evaluated by calculating the ratio of bending moduli (*A*), that is, (*A*_Cell_/*A*_PF_), based on which a theoretical study predicted an *A*_Cell_/*A*_PF_ ratio of ~0.15 for *Leptospira* [29]; the PF is stiffer than the cell body. Another model showed an *A*_Cell_/*A*_PF_ ratio of ~5 for *Borrelia*, which was consistent with the experimental value obtained by stiffness measurements of the borrelial cell body and the PF using optical tweezers [30]; in this case, the PF is stiffer than the cell body. The elastic properties of the cell body and the PF are crucial determinants of species-specific morphology and are thought to be related to the swimming mechanism described later [31].

The filament is connected to the flagellar motor via a hook structure. The hook in *E. coli* consists of the flagellar hook protein (FlgE) and is flexible enough to function as a universal joint to transmit the torque generated by the basal motor to the filament, regardless of the direction [24]. Although the spirochetal hook is also formed by FlgE, *T. denticola* FlgE features self-catalytic intersubunit crosslinking between conserved lysine and cysteine residues, thereby conferring structural stability [32]. The proper stiffness of the hook could be important for the interaction between the PF and the cell body.

### 3.2. Structure of the PF Filament

The *E. coli* flagellar filament is formed by tens of thousands of copies of a single flagellin protein, FliC [24]. Species with more complicated flagella are composed of multiple flagellins, for example, *Campylobacter jejuni* (FlaA and FlaB) and *Caulobacter crescentus* (FljJ, FljK, FljL, FljM, FljN, and FljO) [24]. All spirochete PFs known also consist of more than two proteins, and they generally contain FlaA and FlaB. In *B. burgdorferi*, FlaB forms the entire PF filament, and FlaA is believed to be localized around the base of the filament near the basal motor [27]. The PFs of *B. hyodysenteriae* and *Leptospira* spp. comprise a core filament and sheath [16]. In *B. hyodysenteriae*, three FlaB proteins (FlaB1-3) assemble to form a helical core filament (2.4 μm in wavelength and 0.6 μm in helix diameter), and an FlaA protein assembles to form a straight sheath; association of the FlaB core with the FlaA sheath determines the morphology of the fully assembled PFs (2.8 μm in wavelength and 0.9 μm in helix diameter) [17,18]. Synthesis of the PF and swimming motility in *B. hyodysenteriae* are affected by double knockout of *flaB1*-*flaB2* but not by double knockout of *flaB1*-*flaB3* or single knockout of *flaB3*, highlighting the importance of FlaB1 and FlaB2 in the *Brachyspira* core filament and the possibility of functional compensation between these two proteins [18]. In *Leptospira* spp., PF also consists of the core and the sheath, and six proteins have been identified as PF components: FlaA1, FlaA2, FlaB1, FlaB2, FcpA, and FcpB. PFs isolated from leptospiral cells exhibit a coiled shape [15], but the core filament is straight in the absence of a sheath, indicating that the sheath is indispensable for bending the leptospiral PF [19,21]. The PF core filament of the non-pathogenic species *Leptospira biflexa* is formed by FlaB1 and FlaB2 [19]. The remaining four proteins are involved in synthesizing the sheath or in coiling the PF through core–sheath interactions; however, their roles are not fully elucidated. Deletion of *flaA1* and *flaA2* does not affect the synthesis of the sheath [20], whereas *fcpA* knockout mutants lack a sheath [19,26]. Immunoprecipitations showed the interaction of FcpA with FlaB1 and FlaA2 [19]. These results suggest that FcpA is a major sheath component and plays a central role in coiling via its interaction with the core filament. Recently, cryo-electron microscopy revealed that FcpB is a sheath protein that is localized along the outer curve of the PF, suggesting a contribution to PF coiling [22,23].

### 3.3. Flagellar Motor

Spirochetes and externally flagellated species share fundamental motor parts for rotation, a rotor and a dozen stator units (torque generators) [24], but spirochetes flagellar motor has some spirochete-specific structures, resulting in a unique performance. Motor torque is generated by interaction between the rotor and the stator [33]. Assuming that the force generated by a single stator unit (*F*_S_) is the same among species, the produced motor torque (*M*) depends on the radius of the rotor ring (*r*_R_ ≈ the distance between the motor axis and the rotor-stator contact point) and the number of stator units assembled to the motor (*N*_S_): *M* = *F*_S_ × *r*_R_ × *N*_S_ [34]. Cryo-electron tomography showed that the rotor ring in spirochete motor is larger than that in other external flagellar motors: ~31 nm for *B. burgdorferi*, ~20 nm for *S. enterica*, ~22 nm for *Vibrio fischeri*, and ~27 nm for *C. jejuni* [34]. Thus, the flagellar motor with a larger rotor ring allows more stators to surround the rotor. In addition to the geometrical advantage, the number of assembled stators of externally flagellated species is dynamically altered by changes in load against the motor and the input energy for rotation (e.g., *N*_S_ is decreased up to one near zero load) [24,35,36,37,38], whereas the maximum number of stator units could be incorporated into motors under any conditions in spirochetes [3,39,40,41]. Such stable assembly of the spirochete stators is thought to involve a spirochete-specific motor component called “P-collar” conserved in *T. primitia* [39], *T. pallidum* [41], *B. burgdorferi* [3], *L interrogans*, and *L. biflexa* [40]; perhaps the part plays a key role in stator assembly [34]. This knowledge predicts that the spirochetal motor can produce higher torque, which is supported by motility measurements showing that *Leptospira* spp. produce a stall torque of ~4000 pN nm [10], whereas the stall torque of *E. coli* is ~2000 pN nm [42].

## 4. Swimming Motility

### 4.1. PF-Dependent Swimming

**Figure 2:**
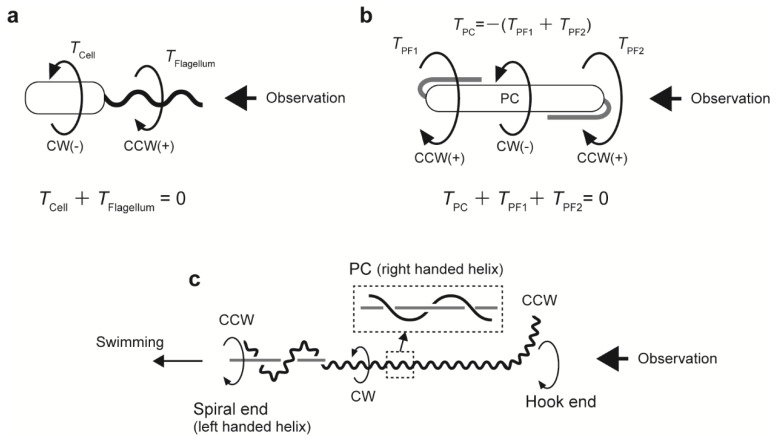
Mechanical models for bacterial swimming. (**a**) Steady-state swimming of an externally flagellated bacterium. Torques of the cell body (*T*_Cell_) and flagellum (*T*_Flagellum_) are balanced, that is, their sum is zero. (**b**) Schematic of spirochetal swimming, where the outer membrane is ignored. The protoplasmic cylinder (PC) is rotated by the counter torque of the periplasmic flagella (PFs) rotating at both ends of the cell body. (**c**) Swimming model for *Leptospira*. Rotational directions are indicated by large arrows.

In externally flagellated bacteria, when viewed from behind a swimming cell, a left-handed helical flagellum rotates counterclockwise (CCW), which is balanced by the clockwise (CW) rotation of the cell body (Figure 2a) [43]. In the case of spirochetes, the protoplasmic cylinder is believed to be rotated in the opposite direction of the PF rotation (Figure 2b) [14]. Rotation of the PFs of *Borrelia* and *Brachyspira* drives wave propagation along the cell body, thus providing thrust for swimming [44]. In contrast, the swimming form of *Leptospira* is more complex. When viewing a swimming *Leptospira* cell from its posterior side, the PF transforms both ends of the cell body into a left-handed spiral or a hook shape and gyrates the bent ends in a CCW fashion; concurrently, the PF rotates the right-handed protoplasmic cylinder in a CW manner (Figure 2c) [11,12]. The majority of thrust for *Leptospira* swimming is given by gyration of the spiral end and rolling of the protoplasmic cylinder [10]. However, correlative speed variation between the protoplasmic cylinder and the hook end was observed [14], suggesting that *Leptospira* swimming depends on mechanical communication among the three rotating parts. 

### 4.2. Energy Input for Spirochete Motility

The bacterial flagellar motor is fueled by the ion motive force (*IMF*), which is the sum of the membrane voltage (*Δψ*) and the ion concentration gap between the cell exterior and interior (*ΔpI*). *E. coli* and *S. enterica* use the proton motive force (*PMF* = *Δψ* + *ΔpH*) for flagellar rotation, whereas *Vibrio cholerae* uses the sodium motive force (*SMF* = *Δψ* + *ΔpNa*) [24]. The coupling ion used in torque generation by the flagellar motor depends on the type of stator units [45]. The MotA/MotB complex present in *E. coli* and *S. enterica* is an H^+^-type stator, and the PomA/PomB complex of *Vibrio* spp. is a Na^+^-type stator. *Vibrio alginolyticus* uses MotA/MotB and PomA/PomB stators for the lateral flagella and polar flagellum, respectively [46,47]. *Bacillus subtilis* also possesses both H^+^-type MotA/MotB and Na^+^-type MotP/MotS complexes [48,49]. Such hybrid stator systems can exchange stator units in response to changes in environmental conditions, such as pH and viscosity [50]. The coupling ion for spirochete motility was investigated in some species by using ionophores and Na^+^ inhibitors, showing that *B. burgdorferi* [51] and *Spirochaeta aurantia* [52] utilize H^+^ for swimming, because they are completely paralyzed by the protonophore carbonyl cyanide m-chlorophenylhydrazone (CCCP). Swimming of *L. biflexa* is also inhibited by CCCP in acidic to neutral pH, while some residual motility is observed under alkaline conditions, even in the presence of CCCP [53]. Moreover, addition of Na^+^ to the medium enhances leptospiral motility [53]. These results suggest the possibility that the major coupling ion for *Leptospira* swimming is H^+^, and that Na is used secondarily in alkaline conditions.

### 4.3. Coordinated Rotation of PFs

The flagellar motor rotates both CCW and CW, and a reversal of the direction of motor rotation results in a change in the swimming direction. In *E. coli*, a rotational switch from CCW to CW unravels the flagellar bundle and thus causes an instant tumbling motion, which is followed by swimming in a randomly determined direction upon returning to CCW rotation [24,33]. Motor reversal from CCW to CW rotation in the polarly flagellated bacterium *V. alginolyticus* changes the swimming direction from forward to backward, whereas the reversal from CW to CCW causes “buckling” of the flagellum at the hook, resulting in a 90 degree change in swimming direction [54]. These motor reversal-based changes in swimming direction are related to bacterial chemotaxis, which may be stimulated by chemicals, temperature, light, and other trigger mechanisms [55]. In spirochetes, rotational directions of PFs are important for directed swimming [6,44]. According to the schematic structure shown in Figure 1a, the flagellar motors residing at both cell ends have to rotate in opposite directions to each other; if they rotate in the same direction, the cell body will not be rotated due to the counterbalance of torques generated by the two motors or the inability to swim due to a twist of the cell body. This mechanical model suggests that asymmetric rotation and synchronized motor reversal between PFs are required for the cells to swim smoothly and change swimming direction [44]. 

Coordinated rotation of *E. coli* flagellar motors can be observed when they reside close to each other, which was explained by diffusion of the phosphorylated chemotaxis response regulator CheY (CheY-P) within the cytoplasm. CheY-P molecules generated in response to methylation of the methyl-accepting chemotaxis protein (MCP) bind to a rotor protein FliM and induce a conformational change of the rotor. As a result, the rotor switch rotation direction from CCW to CW. The delay time of reversal observed between the two motors is consistent with the diffusion time of CheY-P (~100 ms) [56]. CheY is also involved in spirochete chemotaxis [57,58,59,60], but whether its diffusion can manage signal transduction between motors depends on the distance. CheY-P diffusion could be effective in *E. coli* cells that are 1–2 μm in length [56] but not for rapid coordination [61] of spirochete motors that are more than 10 μm apart from each other. Using the equation giving time *t* for diffusing *x* with the diffusion constant *D*, *t* = *x*^2^/2*D*, CheY with a diffusion coefficient of *D* ≈ 10 μm^2^/s [56,62] can be estimated to take 5 s for diffusing 10 μm. This estimation suggests that a CheY-independent mechanism could control the rapid swimming reversal observed in spirochetes. Furthermore, a chemotaxis-deficient *B. burgdorferi* mutant (*cheA* knockout strain) swims straight without reversal, indicating that asymmetric rotation of PFs at different poles of a single cell during steady-state swimming is not related to the chemotaxis system [44]. *B. burgdorferi* possesses two *fliG* homologs, *fliG1* and *fliG2*. FliG1 plays a central role for torque generation through interaction with stator units. FliG2 is essential for PF synthesis in *B. burgdorferi* [63]. Knockout of *fliG1* does not affect PF synthesis, but subcellular localization studies on FliG1 tagged with green fluorescent protein (GFP) revealed that the localization of FliG1 is asymmetric [63]. This suggests the possibility that asymmetric PF rotation observed for *B. burgdorferi* can be attributed to structural differences in flagellar motors residing at both cell ends. Furthermore, a mathematical model predicted the importance of the interaction between PFs at the cell center. In a borrelial model with a single PF, free swimming of the spirochete was reproduced by assuming that both ends of the PF are anchored to the cell body (intimate interaction between PFs) but not by assuming that only one end of the PF is anchored (no interaction between PFs). In the case of *Leptospira* with short PFs, given that the leptospiral cell body is stiffer than PFs [29], torque transmission from one end to the other may occur along the cell body instead of being mediated by direct contact between PFs.

### 4.4. Translation Versus Rotation

**Figure 3:**
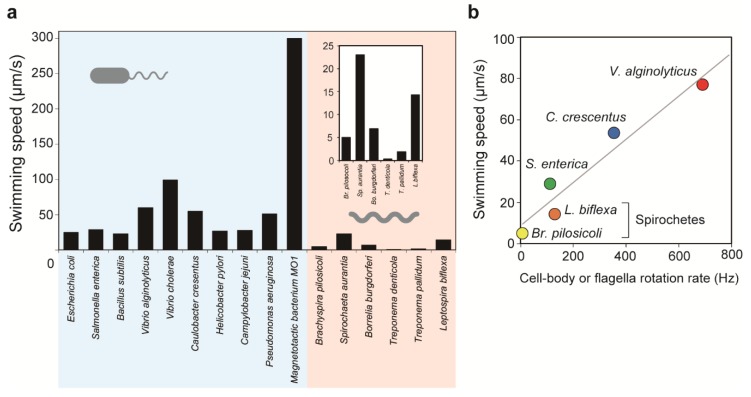
Speeds of bacterial motility. (**a**) Swimming or gliding speeds of various bacterial species. Spirochete-derived data are enlarged in the inset. Refer to the following literature for the corresponding swimming measurements: *E. coli* [65], *S. enterica* [74], *B. subtilis* [49], *V. alginolyticus* [75], *V. cholerae* [67], *C. crescentus* [66], *Helicobacter pylori* [76], *C. jejuni* [77], *Pseudomonas aeruginosa* [78], magnetotactic bacterium MO-1 [68], *B. pilosicoli* [8], *S. aurantia* [79], *B. burgdorferi* [70], *T. denticola* [80], *T. pallidum* [71], and *L. biflexa* [10]. (**b**) Relationships between rotation rates and swimming speeds: *S. enterica* [64], *V. alginolyticus* [72], *C. crescentus* [81], *B. pilosicoli* [8], and *L. biflexa* [10].

Swimming speeds differ significantly among species (Figure 3a). *E. coli* and *Salmonella* spp. swim at 20–30 μm/s [64,65], while *C. crescentus* (~60 μm/s) [66], *V. cholerae* (~100 μm/s) [67], and the magnetotactic marine bacterium MO-1 (~300 μm/s) [68] are examples of faster swimmers. In comparison with externally flagellated bacteria, the swimming speed of spirochetes in liquid media is much slower. The fastest swimmer is *Leptospira* spp. (~15 μm/s) [10,69], which is followed by *B. burgdorferi* (~7 μm/s) [70], *Brachyspira pilosicoli* (~5 μm/s) [8], and *Treponema pallidum* (~2 μm/s) [71]. Swimming speeds are correlated with cell body rotation rates or wave frequencies (Figure 3b). Dividing the swimming speed *v* by the rotation rate or the wave frequency *f* gives the migration distance achieved by one revolution of the helical body, that is, *v*/*f*. The ratio of *v*/*f* to helix pitch *p*, (*v*/*f*)/*p*, is similar to motion efficiency; for example, equal values of *v*/*f* and *p*, that is, (*v*/*f*)/*p* = 1, indicate swimming without slip [72]. The (*v*/*f*)/*p* ratios of *S. enterica* and *V. alginolyticus* are ~0.1 [64] and ~0.07 [72], respectively, meaning that these bacteria move by less than 10% of the helix pitch of their flagella by one flagellar revolution. *B pilosicoli* and *L. biflexa* show (*v*/*f*)/*p* values of ~0.17 [8] and ~0.27 [73], respectively, showing slightly more efficient swimming than external flagella-driven motility. Spirochetal (*v*/*f*)/*p* values increase with viscosity, leading to increased swimming speeds at high viscosity (described below).

### 4.5. Effect of Viscosity on Swimming Motility

Although the swimming ability of spirochetes seems to be inferior to that of other flagellated bacteria (Figure 3), spirochete swimming is known to be improved by increased viscosity. Kaiser and Doetsch reported that the swimming speed of *L. biflexa* monotonically increased with viscosity in methylcellulose solutions [82]. Similar phenomena have been observed in *B. burgdorferi* [83], *T. denticola* [80], and *B. pilosicoli* [8]. *T. denticola* cannot swim at all in medium without polymers, but smooth translation is allowed by the addition of methylcellulose to the medium (~6 μm/s in 1% methylcellulose 4000 solution) [80]. However, swimming motilities of these spirochetes cannot be improved by all types of viscous fluids but only by gel-like, heterogeneous polymer solutions, for example those containing methylcellulose, polyvinylpyrrolidone (PVP), or mucin [8,69,83,84]. These linear polymers form a quasi-rigid network and are thus treated as viscoelastic fluids [85]. In contrast, the swimming speeds of *B. pilosicoli* [8], *L. biflexa* [10], and *B. burgdorferi* slow down in the presence of the branched polymer Ficoll that does not form a network [71]. Measurements in *B. pilosicoli* highlighted that the *v*/*f* value of this spirochete was improved by addition of PVP but not Ficoll [8]. Although the mechanisms by which spirochete motilities are influenced by the differences in microscopic polymer structure are not fully understood, viscoelasticity is believed to be related to this unique phenomenon.

**Figure 4:**
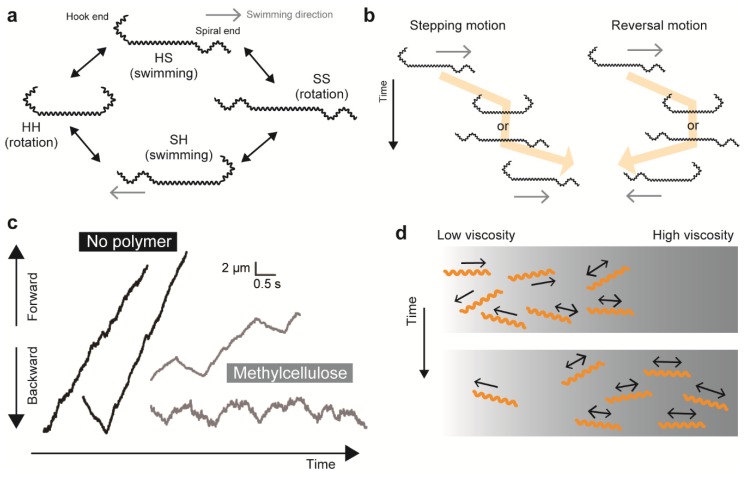
Effect of viscosity on *Leptospira* swimming. (**a**) Association of cell morphology and swimming in *Leptospira*. The spirochete can swim while displaying asymmetric morphologies (SH or HS), with the front end pointing towards the swimming direction and usually displaying a spiral shape. (**b**) Definition of stepping and reversal motions. (**c**) Reversal movements are enhanced by the addition of methylcellulose to the medium. (**d**) A plausible explanation of “viscotaxis” in *Leptospira*. Enhanced swimming reversal with elevated viscosity suppresses net migration of *Leptospira* cells, facilitating an accumulation of spirochetes in high viscosity areas.

*Leptospira* are known to be attracted to higher viscosity, and the mechanism of this so-called “viscotaxis” was explained by the viscosity-dependent increment of swimming speed [86]. However, a recent motility study using *Leptospira* proposed another plausible model of taxis-like behavior, which was based on the result that a change in viscosity affects the reversal frequency in swimming direction [13]. When a leptospiral cell swims with the anterior spiral (S) end and the posterior hook (H) end (SH form), the transformation into symmetric cell morphology (SS or HH form) interrupts swimming transiently, although the cell keeps rotating (Figure 4a). Leptospiral swimming is restarted by transformation from symmetric to asymmetric forms, and the swimming direction after exhibiting symmetric morphologies is determined by the cell forming SH or HS. The transformation process of SH-SS/HH-SH causes a pause of swimming but does not change the swimming direction (stepping movement), whereas SH-SS/HH-HS turns the swimming direction by 180 degrees (reversal movement) (Figure 4b) [13]. Takabe et al. measured the stepping and the reversal events of individual leptospiral cells in various viscous solutions containing methylcellulose, Ficoll, or the major viscous agent for tissue mucin, showing that the reversal frequency increased with viscosity (Figure 4c) [13]. The reversal movement returns the cell to its original position, indicating that there is no net migration. Thus, viscosity-dependent impairment of net migration occurs due to the increment of the reversal event that results in trapping leptospires in areas with higher viscosity, which could assist the accumulation of spirochetes in the mucus layer in vivo (Figure 4d).

## 5. Chemotaxis

Early studies on chemotaxis using *E. coli* and *S. enterica* showed that these are attracted to nutritious substrates, such as sugars and amino acids, but are repelled by harmful ones, such as alcohols. Notably, not all of the attractants and repellants are related to metabolism [87,88]. In spirochetes, *S. aurantia* shows an attraction response to many sugars, such as glucose, xylose, galactose, and fructose [79], whereas *B. hyodysenteriae* is attracted to serine, fucose, and lactose [89]. *B. burgdorferi* does not respond to common chemicals, such as sugars and amino acids, but is attracted to rabbit serum and is repelled by ethanol and butanol [51]. Both pathogenic and saprophyte *Leptospira* spp. are attracted not only to their sole carbon sources, i.e., long-chain fatty acids, but also to sugars (e.g., glucose) that cannot be metabolized in *Leptospira* [90,91,92]. Chemotaxis to hemoglobin was observed in the pathogenic species *L. interrogans* but not in saprophytes [93]. 

Chemotaxis is closely related to the reversal of flagellar rotation, as described in Section 4.3. Motor reversal in peritrichous bacteria results in an exploration of the environment by repeated run-and-tumble movements [24,33] and causes back-and-forth movements with ~90 degree changes in swimming direction by buckling in the case of polarly flagellated bacteria [54]. The swimming pattern of spirochetes involves back-and-forth motions, and attractants increase the persistency of their directed runs [91]. However, when swimming freely in liquid medium, the spirochetal back-and-forth movement cannot result in changes in direction as large as *Vibrio*, because the spirochete cell body is elastic but not too flexible to be buckled by mechanical stress. A physical study on *Leptospira* showed that such a long and spiral body has a larger diffusion coefficient than a simple rod, suggesting that the exploration of spirochetes involves passive Brownian motion in addition to active swimming [94].

## 6. Movement on Solid Surfaces

**Figure 5:**
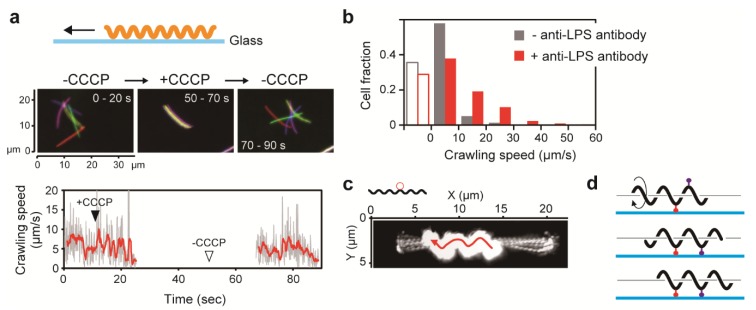
Crawling motility of *Leptospira*. (**a**) Effect of carbonyl cyanide m-chlorophenylhydrazone (CCCP) on *Leptospira* crawling on a glass surface. (**b**) Effect of anti- lipopolysaccharide (LPS) antibody on crawling speed. Open bars indicate the fractions of cells adhered to the glass without crawling. (**c**) Movement of a microbead coated with anti-LPS antibody on the leptospiral cell surface. Sequential frames of a movie were superimposed to show the bead trajectory. (**d**) Schematic explanation of crawling. Adhesive molecules (red and purple symbols), such as LPS, anchor the cell to a surface, and PF-dependent rolling of the protoplasmic cylinder propels the cell.

*Pseudomonas aeruginosa* not only swim with a polar flagellum but can also move on a solid surface using pili in a process called twitching motility [2,95]. To that effect, ambivalent motility of *P. aeruginosa* is realized by two distinct machineries specialized for movement in liquid and on solid media, respectively. A major motility form of spirochetes is swimming, but *Leptospira* spp. can move both in liquid and on solid surfaces. Cox and Twigg first reported leptospiral snake-like movement on a smooth surface, which was called “crawling” [96]. For moving while attached to surfaces, *Mycoplasma mobile* uses abundant leg-like protein complexes that are expressed on the cell surface; these legs successively catch and release sialylated oligosaccharides on surfaces, thereby propelling the cell [97]. Another gliding bacterium, *Myxococcus xanthus*, has a machinery that is composed of intracellular motor proteins and an external adhesive complex (Agl-Glt) [98]. Leptospiral swimming is a result of flagella-dependent motility, but a machinery specialized in crawling has yet to be identified. Charon et al. observed that microbeads attached to the leptospiral cell surface via anti-whole cell antibody freely move along the cell body, suggesting that unspecialized antigens residing on the outer sheath are involved in crawling motility by functioning as mobile adhesins [99]. A recent study by Tahara et al. showed that crawling is completely inhibited by CCCP, indicating that PMF-dependent PF rotation drives crawling (Figure 5a) [73]. Furthermore, it was revealed that modification of glass surfaces with anti-lipopolysaccharide (LPS) antibody affects the crawling speed and that anti-LPS antibody-coated microbeads move on the outer bacterial membrane. These results suggest that LPS is responsible for crawling, serving as one of the adhesins anchoring the cell to the surface (Figure 5b–d) [73]. Electron microscopic observation of a hamster liver infected by pathogenic leptospires showed entry of leptospiral cells into the intercellular junction of hepatocytes [100], implying that leptospiral pathogenicity could involve adherence of spirochetes to host cells, followed by crawling (discussed in Section 7).

## 7. Motility as A Virulence Factor

In general, bacterial flagella and motility are related to virulence, such as invasion, adhesion, and others [101,102]. Motility is an essential virulence factor for pathogenic spirochetes, and loss of motility due to a lack of flagellar genes attenuates infections with *B. burgdorferi* [63], *B. hyodysenteriae* [103], and *L. interrogans* [20,21]. Invasion of *B. burgdorferi* via a tick bite induces a hallmark rash, called erythema migrans, at the initial stage of Lyme disease. Motility analyses of *B. burgdorferi* using the mouse dermis showed three distinct motilities of the spirochete, which were termed translocating, wriggling, and lunging [70]. The translocating state is similar to swimming in solutions, whereas the wriggling (the entire cell body is fixed in place but keeps undulation) and the lunging (the cell body is partially fixed on the surface) states are observed only in the dermis or the gelatin resembling the mouse dermis. The translocation is essential for dissemination within the host, and transient adhesion by wriggling and lunging is thought to be involved in changing the moving direction and evading host immune system [70]. *Brachyspira* spp. penetrate the epithelial mucosa with one end of the cell body moving in the same direction, and this well-aligned colonization is called “false-brush-border”, which could involve directed motility of spirochetes [104]. In *Leptospira* spp., pathogenic strains are classified into ~300 serovars based on the structural difference in LPS, and the severity of the infection outcome depends on the combination of host species and leptospiral serovars [105]. Although the details on the relationship between motility of *Leptospira* serovars and their host-dependent pathogenicity remain unknown, the crawling motility mediated by leptospiral LPS and other adhesion molecules is a potential key factor [73,106]. Recently, we measured adhesivity and crawling of some leptospiral serovars on kidney cells derived from various mammalian hosts, including humans, showing close correlation of the measured parameters with the symptom severity of the host–serovar pairs; pairs causing more severe symptoms, such as hemorrhage, jaundice, and nephritis, show high adhesivity and persistent crawling of leptospires on the host cells [106]. This knowledge is an important step toward understanding the host–pathogen relationship to develop novel antimicrobials for targeting pathogen dynamics.

## 8. Conclusions and Perspectives

Members of the spirochetes share a basic cell structure, but their configurations, PF compositions, and motility forms are extremely diverse. Remarkable advancements in cryo-electron microscopy/tomography have unveiled many spirochete-specific structures, such as the motor scaffold P-collar, fully assembled stator units, and a combination of multiple proteins for establishing the unique morphology of PFs. These are important clues to discuss high torque generation by the spirochetal flagellar motor. Motility measurements by optical microscopy showed improved efficiency of swimming motility in gel-like fluids and viscosity-dependent enhancement of swimming reversal, probably facilitating an accumulation of spirochetes in viscous milieus that exist abundantly within a host body. A recent study showed the close relationship of the spirochetal movements over host cell surfaces and the severity of the symptoms caused, giving crucial insight into the practical role of bacterial motility as a virulence factor.

Although the knowledge summarized in this review deepened the understanding of the mechanics of spirochete motility and its biological significance, there are still many issues remaining, such as the interaction between spirochetes and viscoelastic fluids, signal transduction for the coordinated rotation of PFs between both cell ends, and the molecular basis of crawling motility on the host cells. Further studies on these subjects will advance biomimetic technology and prompt the development of novel prevention/medication strategies.

## Figures and Tables

**Table 1:** Comparison of the cell structure and the periplasmic flagella (PFs) among three spirochete species.

Species (Disease)	Cell Morphology		Cell Body Parameters			PF				Ref.
			Length	Width	Wavelength	Number	Shape	Overlap	Proteins	
*Borrelia burgdorferi* (Lyme disease)	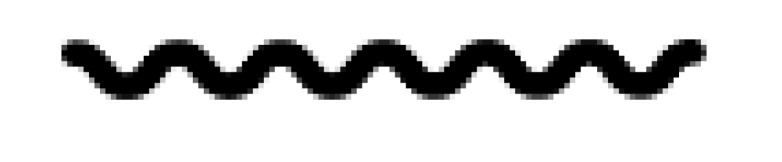	**Flat wave**	~20 μm	~0.3 μm	~2.8 μm	14~22	Left-handed helix	Yes	FlaA FlaB	[4,5,6,7]
*Brachyspira hyodysenteriae* (Swine dysentery)	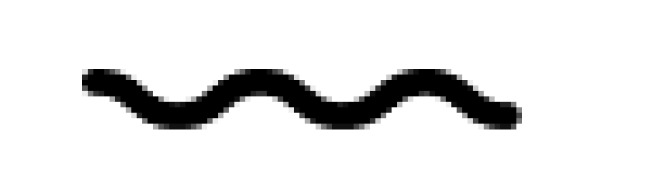	Flat wave?	~10 μm	~0.3 μm	~4 μm	16~18	Left-handed helix	Yes	FlaA FlaB1,2,3	[8,16,17,18]
*Leptospira interrogans* (Leptospirosis)	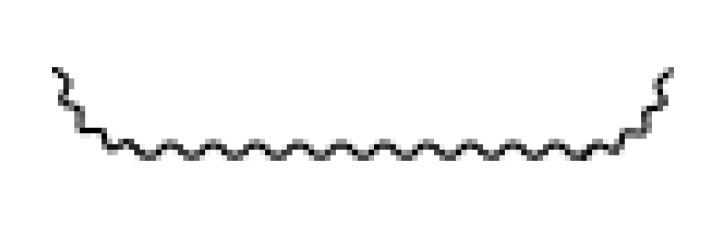	Right-handed helix	~20 μm	~0.15 μm	~0.7 μm	2	Coiled shape	No	FlaA1,2 FlaB1,2 FcpA, FcpB	[4,10,15,19,20,21,22,23]
